# Association between maternal interpregnancy interval after live birth or pregnancy termination and birth weight: a quantile regression analysis

**DOI:** 10.1038/s41598-018-22498-0

**Published:** 2018-03-07

**Authors:** Qi Zhang, Shaonong Dang, Ruhai Bai, Baibing Mi, Lingling Wang, Hong Yan

**Affiliations:** 1 Department of Epidemiology and Biostatistics, School of Public Health, Xi’an Jiaotong University Health Science Center, Xi’an, Shaanxi Province P.R. China; 2 Global Health Institute of Xi’an Jiaotong University Health Science Center, Xi’an, Shaanxi Province P.R. China; 3 Nutrition and Food Safety Engineering Research Center of Shaanxi Province, Xi’an, Shaanxi Province P.R. China

## Abstract

We used quantile regression (QR) to assess if the length of the interpregnancy interval (IPI) after live birth and pregnancy termination is associated with weight in subsequent birth. The analysis included 9663 and 3400 women with IPI after live birth and pregnancy termination, respectively. For the women after live birth, an IPI < 12 months had negative effects at the 5th and 10th quantiles of the birth weight (BW) distribution. When the BW was beyond the 90th quantile, the BWs of newborns whose mothers with longer IPI (36–59 months) were higher than the reference group (18~23 months). For women after pregnancy termination in the 10^th^ quantile, it was observed that those pregnant women with IPIs between 36 and 47 months had a negative effect (150 g) on BW compared with the reference group. This finding revealed that mothers with IPI < 12 months resulted in a decrease of 85 g at 75th quantile. The impact of IPI > 119 months in the upper quantile (95^th^) had an increase of 330 g in BW. Our results demonstrated that both short (<12 months) and long (>36 months) IPIs are independently associated with higher risks of low birth weight (LBW) and macrosomia.

## Introduction

The birth weight (BW) of newborns serve as one of the major determinants for physical and mental growth in infants and also reflects the intrauterine growth of the fetus^1^. Previous studies have shown that both low birth weight (LBW) and macrosomia increase the risk of adverse pregnancy outcomes and subsequent health problems in the offspring^2^. LBW is associated with increased morbidity and mortality in infancy and adolescence^3^. Macrosomia is also related to poor health outcomes for the child and mother, with an increased risk of neonatal hypoglycemia, cesarean section, postpartum hemorrhage, shoulder dystocia, longer hospitalization and metabolic syndrome in the offspring with a predisposition to develop cardiovascular disease and diabetes in adulthood^4,5^.

The interpregnancy interval (IPI) is defined as the time that has elapsed between the date of the previous delivery and the first day of the last normal menstrual period for the index pregnancy^6^. The IPI is considered to be a crucial and modifiable risk factor for adverse birth outcomes^7–10^. The incidences of small for gestational age birth (SGA), preterm birth and LBW have each been repeatedly shown to follow a strong J-shaped relationship to the IPIs^7,8^. Compared with intermediate intervals of 18–23 months, short intervals (<18 months between the previous birth and subsequent conception) and long intervals (>23 months) are associated with a higher risk of adverse birth outcomes^7–9,11^. Although the above mentioned studies demonstrated a valuable relationship between the IPI and BW, the results do not completely elucidate the relationship between explanatory and outcome variables. The use of quantile regression (QR) to estimate the association between IPI and BW has been limited based on the extant literature. The present study therefore intended to overcome this limitation. The reported models, whether designating BW as the dependent variable or logistic regression keeping LBW or macrosomia as the dependent variable, only describe the conditional mean of an outcome without describing the scale of the distribution and the classification also lead to a loss of information^6,12^. The QR approach we utilized in this paper, which provides a complete image of the effects of covariates on BW by estimating the family of conditional quantile function^13^, makes QR a natural choice for this analysis because of the robust property and lack of distribution hypothesis.

As a consequence, the purpose of our study was to characterize the effect of IPIs along the entire BW distribution using the data from a population-based survey by the quantile regression in order to provide valuable policy insight to some extent.

## Methods

### Study design and subjects

The data in the current study were derived from a population-based, cross-sectional survey conducted between August and October 2013. The subjects were Chinese women of childbearing age (15–49 years) living in Shaanxi, a major province in western China. Shaanxi has a population of 40 million and is undergoing rapid economic development. The inclusion criteria for the women were a history of pregnancy between August 2011 and August 2013 and a gestational age ≥28 weeks. Women were excluded if they were pregnant during the time of the survey or had a serious illness, such as cancer or cardiovascular disease. A stratified multi-stage sampling method was adopted to obtain the representative sample based on the proportion of urban and rural populations and fertility rates. First, the entire population of women of childbearing age was stratified into urban and rural strata. 20 counties and 10 districts were randomly selected from the two strata. Second, 6 townships from each chosen county and 3 neighborhoods from each chosen district were randomly sampled. Third, 6 villages from each township and 6 communities from each neighborhood were randomly sampled. Finally, 30 eligible women from each selected village and 60 eligible women from each selected community were randomly sampled.

### Data sources

Face-to-face interviews with a structured questionnaire were conducted by trained interviewers after obtaining written informed consent. The additional child level information, such as BW and birth date, were obtained through a review of birth certificates and the BW data were corrected to the nearest 10 g. Data involving the women’s reproductive history were obtained by asking about previous pregnancy outcomes, including miscarriages, induced abortions, BW, gestational age and birth or termination date associated with previous pregnancies. We considered all early pregnancy losses and stillbirths and terminations of pregnancy that occurred after 28 weeks gestation. The supervisors reviewed each questionnaire immediately after completion for missing values or logic errors to assure the accuracy of the data. The pediatrician from each team helped investigators gather information about birth outcomes.

There were 30,027 registered pregnancies during this period. After excluding non-live births (n = 761), unknown outcomes (n = 350), primigravidas (n = 14402) and women with incomplete reproductive histories (n = 1375), the final data set consisted of 13,063 cases with an IPI.

### Study variables

In this research, BW was considered to be the main outcome (dependent variable). The IPI, the independent variable, was calculated as the time between the previous live birth or pregnancy termination and the present live birth. The IPI was calculated from the previous live birth and termination dates. From a practical point of view, the classification of IPI might be better for understanding the effect of a shorter or longer IPI on BW. Therefore, the IPI was categorized in the current study. To be consistent with previous studies^7,14,15^, the IPI was classed as <12, 12~17, 18~23, 24~35, 36~47, 48~59, 60~119 and ≥120 months. We used an IPI of 18~23 months as the reference. The BW refers to the weight of newborns within 1 h after birth, which was obtained from the birth certificate.

### Covariates

Some factors, including demographic factors, have been suggested to be associated with BW^16–18^. These factors were defined as covariates in our study to illuminate the relationship between the IPI and BW. The covariates included residence (urban or rural), infant gender (male or female), gestational age in weeks (<37, 37~41 and >41), maternal age in years (<20, 20~35 and >35), maternal education in years (<9, 9 and >9), ethnicity (Han and others), gravidity (2 and >2), maternal job (employee or non-employee), pregnancy-induced hypertension (PIH; yes or no) and family economic status. Gestational age was computed as the number of completed weeks of gestation from the date of the mother’s last menstrual period-to-delivery. Family economic status referred to the per capita annual household income and was divided into low, middle and high by quartile. The information on covariates was obtained from the baseline questionnaire.

### Statistical analysis

The data were manually checked for completeness and double-entered into EpiData version 3.1 (EpiData Association, Denmark). Mean ± standard deviation (SD) was used for the description of continuous variables and categorical variables are presented as counts and percentages. Simple linear regression was used for univariate analysis. We compared sociodemographic data, maternal pregnancy and birth-related characteristics of the present live birth by previous pregnancy outcome and we also compared the BW between these characteristics. Significant differences were defined as comparisons with a *P* value < 0.05 and a 95% confidence interval, not inclusive of the null value of 0.0.

We employed OLS regressions and QRs to assess associations of predictor variables with the mean and the 5^th^, 10^th^, 25^th^, 50^th^, 75^th^, 90^th^ and 95^th^ quantiles of the BW. The potential modifying effect of IPIs on BW was further adjusted for covariates, including residence, infant gender, gestational age, maternal age, maternal education, ethnicity, gravidity, maternal job, PIH and economic status. We adjusted for urban-rural stratification.

QR as a location model was proposed by Koenker and Bassett^19^ to extend OLS, which generalizes the distribution at its mean to a more general class of linear models in which the conditional quantiles have a linear form to fully account for the overall distribution of the response variable. To formalize the QR, a real valued random, variable Y, was characterized by the following distribution function:

1 $${\rm{F}}({\rm{y}})=\Pr ({\rm{Y}}\le {\rm{y}})$$

Then, for any Tɛ(0, 1), the T-th quantile of Y was defined as follows:

2 $${\rm{Q}}({\rm{T}})={\rm{in}}\,{\rm{f}}\{{\rm{y}}:{\rm{F}}({\rm{y}})\ge {\rm{T}}\}$$

The most useful quantiles are often determined by the sample size and subject-matter considerations. The frequently used quantiles, T, from equation (2) were T = 0.25, T = 0.50 and T = 0.75 for the first, median and third quartile, respectively. Thus, unlike the OLS, which minimizes the squared differences around the mean, QR minimizes the weighted absolute difference between the observed value of y and the T-th quantile of Y^19^. Thus, any value between 0 and 1 regulates the regression plane position and the steering, which can lead to an independent estimation of different quantiles of the dependent variable. It not only can represent all of the information in the data, but can also focus more on specific regions of the data, such as extreme position data, instead of just focusing on the conditional mean of the response variable, as does OLS^19^.

Unlike OLS, QR provides a complete view of the influence of an independent variable on the outcome variable. Therefore, it is feasible to identify the more vulnerable groups and devise more effective interventions. Moreover, OLS may be inefficient if the errors are highly non-normal and QR is more robust to non-normal errors and outliers^20,21^. We were also interested in the differences between OLS and QR estimates. The results from OLS and QR were revealed in tables to depict the levels of estimates produced by the two types of estimations.

Statistical analyses were performed using statistical package for SAS version 9.4 (SAS Institute, Cary, NC, USA).

### Ethics Statement

The study was conducted in accordance with the Declaration of Helsinki and was approved by the Human Research Ethics Committee of the Xi’an Jiaotong University Health Science Center (No. 20120008). The approval was accepted on 6 March 2012. Written informed consent was obtained from each student before the questionnaire survey.

## Results

### Background information of subjects

There were 13,063 women with reproductive histories between 2010 and 2013 considered for analysis, with 9663 after a live birth and 3400 after a pregnancy termination (Table 1). Among the women after a live birth, 99.49% (9614) were Han Chinese and more than four-fifths (89.68%) of the women lived in rural areas. Then mean age at delivery was 30.97 years (SD, 4.92), with women 20–35 years of age at delivery accounting for 80.11% of the sample. The majority of participants (78.39%) had completed 9 years of compulsory education (junior high school or above). Most participants (77.76%) were not employed outside the home and were categorized as farmers. The average annual household income per capita was 5075, 8889 and 13,750 yuan in the lower, median and upper quartiles, respectively (Table 1).

**Table 1:** Sociodemographic, maternal pregnancy and birth-related characteristics comparing infant birth weight.

Characteristics	After live birth (n = 9663)				After termination (n = 3400)			
	n (%)	BW (g,$\bar{{\rm{x}}}$ ± s)	*β*	*p*-value	n (%)	BW (g, $\bar{{\rm{x}}}$±s)	*β*	*p*-value
**Residence**								
Rural	8666(89.68)	3259.74 ± 467.09	−93.09	<0.0001	2641(77.68)	3281.30 ± 484.78	−73.49	0.0002
Urban (Ref.)	997(10.32)	3352.83 ± 458.79			759(22.32)	3354.80 ± 460.41		
**Infant gender**								
Female	4170(43.15)	3200.74 ± 457.90	−120.68	<0.0001	1519(44.68)	3226.70 ± 486.54	−128.36	<0.0001
Male (Ref.)	5493(56.85)	3321.42 ± 467.28			1881(55.32)	3355.06 ± 486.54		
**Gestational age**								
<37 weeks	254(2.63)	2713.46 ± 694.77	−568.64	<0.0001	111(3.26)	2628.73 ± 649.01	−686.30	<0.0001
37 to 41 weeks (Ref.)	9203(95.24)	3282.10 ± 449.05			3201(94.15)	3315.03 ± 452.66		
>41 weeks	206(2.13)	3384.93 ± 480.94	102.83	0.0014	88(2.59)	3511.59 ± 563.36	196.56	<0.0001
**Maternal age**								
<20 years old	123(1.27)	3229.67 ± 465.17	−49.16	0.2465	46(1.35)	3380.43 ± 340.83	74.59	0.2956
20 to 35 years old (Ref.)	7741(80.11)	3278.83 ± 461.08			2907(85.50)	3305.85 ± 470.98		
>35 years old	1799(18.62)	3231.23 ± 490.44	−47.61	<0.0001	447(13.15)	3236.29 ± 544.54	−69.56	0.0044
**Maternal education**								
Primary school or below	2089(21.62)	3215.77 ± 500.24	−57.69	<0.0001	397(11.68)	3201.38 ± 474.23	−74.81	0.0051
Junior high school	5838(60.42)	3273.46 ± 456.52			1652(48.59)	3276.20 ± 491.46		
Senior high school or above (Ref.)	1736(17.97)	3319.98 ± 454.43	46.52	0.0003	1351(39.74)	3352.33 ± 461.85	76.13	<0.0001
**Ethnicity**								
Minority	49(0.51)	3142.45 ± 465.45	−127.54	0.0566	38(1.12)	3359.21 ± 437.72	62.20	0.4275
Han (Ref.)	9614(99.49)	3269.99 ± 467.02			3362(98.88)	3297.01 ± 480.83		
**Gravidity**								
>2 times	1176(12.17)	3272.94 ± 497.05	4.09	0.7784	1760(51.76)	3305.67 ± 489.92	16.50	0.3169
2 times (Ref.)	8487(87.83)	3268.85 ± 462.80			1640(48.24)	3289.17 ± 469.87		
**Maternal job**								
Homemaker or farmer	7514(77.76)	3259.53 ± 468.61	−44.13	0.0001	2188(64.35)	3272.06 ± 482.44	−71.97	<0.0001
Employee (Ref.)	2149(22.24)	3303.66 ± 460.13			1212(35.65)	3344.02 ± 473.24		
**PIH**								
Yes	157(1.62)	3103.69 ± 601.88	−168.48	<0.0001	80(2.35)	3044.75 ± 596.27	−259.06	<0.0001
No (Ref.)	9506(98.38)	3272.08 ± 464.06			3320(97.65)	3303.81 ± 475.67		
**Economic status**								
Low (lowest 25)	2014(20.84)	3232.38 ± 458.11	−30.05	0.0134	588(17.29)	3258.26 ± 509.51	−29.48	0.2040
Middle (medium 50) (Ref.)	5482(56.73)	3262.43 ± 468.70			1566(36.65)	3287.74 ± 485.35		
High (top 25)	2167(22.43)	3321.19 ± 458.11	58.76	<0.0001	1246(36.65)	3328.86 ± 457.82	41.12	0.0240

After a pregnancy termination, the overall mean [standard deviation (SD)] age was 29.55 (5.00) years. At delivery, 85.50% (2907) were 20–35 years of age, 99.49% (9614) were Han Chinese, 60.27% (2049) had ≤9 years of education and 77.68% (2641) were from rural areas. Of the women, 64.35% (2188) had occupations related to farming or homemaking. The average annual household incomes per capita were 11000 yuan (Table 1).

### BW by selected factors among the two groups

Univariate analysis showed that there were eight factors associated with BW (Table 1). Infant gender, gestational age, residence, maternal age, maternal education, maternal job and economic status were the demographic factors. Complications, such as PIH, had a significant influence on BW. The results indicated that female neonates, preterm infants, order mothers, low level of maternal education, rural neonates, mothers with no income, mothers with PIH and low economic status had significantly lower BWs than the reference group.

### Distribution of IPI

Among the women after a live birth, 8.91% and 7.13% had IPIs < 12 or <18 months, respectively and 30.44% and 14.60% had IPIs > 59 or >119 months, respectively. Among the women after a pregnancy termination, 41.56% and 12.91% had IPIs < 12 or <18 months, respectively. Distribution of the IPI was positively skewed. After live birth or pregnancy termination, the median interval was 53.77 and 15.58 months, respectively.

### Association between IPI and BW

Tables 2 and 3 gave a comparison of the relationship between different classifications of IPIs and BW using QR and the OLS method for women with a previous live birth or pregnancy termination. The QR coefficients revealed that IPIs for the BW of neonates had different effects at different parts of the distribution.

**Table 2:** QR results for assessing the relationship between different IPI and birth weight for the women after a live birth.

IPI	Coeff (p-value) (95% CI)							
	Q05	Q10	Q25	Q50	Q75	Q90	Q95	OLS
<12 months (n = 861)	−114.29(0.0096) (−200.71~−27.86)	−92.86(0.0222) (−172.46~−13.25)	−50.00(0.1551) (−118.93~18.93)	−43.75(0.971) (−95.43~7.93)	−28.57(0.3339) (−86.53~29.39)	0.00(1.0000) (−92.87~92.87)	0(1.0000) (−109.99~109.99)	−44.12(0.0563) (−89.43~1.19)
12~17 months (n = 689)	0.00(1.0000) (−91.07~91.07)	−14.29(0.7385) (−98.18~69.60)	0.00(1.0000) (−72.64~72.64)	−6.25(0.822) (−60.71~48.21)	−25.00(0.5666) (−43.22~78.94)	50.00(0.3166) (−47.87~147.87)	0(1.0000) (−115.91~115.91)	−1.30(0.9573) (−49.05~46.45)
18~23 months (n = 681)	Ref.							
24~35 months (n = 1139)	−42.86(0.3036) (−124.51~38.80)	−21.43(0.5765) (−96.64~53.78)	0.00(1.0000) (−65.12~65.12)	−6.25(0.8019) (−55.08~42.58)	7.14(0.7982) (−47.62~61.91)	0.00(1.0000) (−87.75~87.75)	50(0.3456) (−53.92~153.92)	−0.86(0.9684) (−43.67~41.94)
36~47 months (n = 1003)	14.29(0.7382) (−69.48~98.05)	28.57(0.4679) (−48.59~105.73)	0.00(1.0000) (−66.81~66.81)	50.00(0.0504) (−0.09~100.09)	53.57(0.0616) (−2.61~109.75)	100.00(0.0295) (9.98~190.02)	50(0.3579) (−56.61~156.61)	52.25(0.0197) (8.34~96.17)
48~59 months (n = 938)	7.14(0.8692) (−77.87~92.16)	42.86(0.2834) (−35.45~121.16)	50.00(0.1484) (−17.80~117.80)	50.00(0.0539) (−0.84~100.84)	42.86(0.1407) (−14.16~99.87)	100.00(0.0319) (8.64~191.36)	150(0.0066) (41.80~258.20)	61.69(0.0067) (17.12~106.26)
60~119 months (n = 2941)	−7.14(0.8466) (−79.53~65.24)	0.00(1.0000) (−66.68~66.68)	0.00(1.0000) (−57.73~57.73)	43.75(0.0476) (0.46~87.04)	25.00(0.3128) (−23.55~73.55)	50.00(0.2077) (−27.79~127.79)	50(0.2874) (−42.13~142.13)	25.55(0.1871) (−12.41~63.50)
>119 months (n = 1411)	−64.29(0.1586) (−153.66~25.08)	−78.57(0.0614) (−160.89~3.75)	−50.00(0.1692) (−121.28~21.28)	31.25(0.2517) (−22.19~84.69)	28.57(0.3501) (−31.37~88.51)	50.00(0.3075) (−46.04~146.04)	50(0.3889) (−63.74~163.74)	1.80(0.9399) (−45.05~48.66)

OLS: ordinary least squares; Q: quantile;

Adjust covariates: Residence, Infant gender, Gestational age, Maternal age, Maternal education, Ethnicity, Gravidity, Maternal job, PIH and Economic status.

**Table 3:** QR results for assessing the relationship between different IPI and birth weight for the women after a pregnancy termination.

IPI	Coeff (p-value) (95% CI)							
	Q05	Q10	Q25	Q50	Q75	Q90	Q95	OLS
<12 months (n = 1413)	−10.00(0.8464) (−111.21~91.21)	−50.00(0.2285) (−131.39~31.39)	−25.00(0.4172) (−85.40~35.40)	−50.00(0.1762) (−122.46~22.46)	−85.00(0.0274) (−160.51~−9.49)	−44.00(0.3163) (−130.07~42.07)	−75.00(0.2037) (−190.67~40.67)	−47.89(0.0980) (−104.62~8.83)
12~17 months (n = 439)	20.00(0.742) (−99.12~139.12)	0.00(1.0000) (−95.79~95.79)	0.00(1.0000) (−71.09~71.09)	−50.00(0.2504) (−135.28~35.28)	−85.00(0.0608) (−173.87~3.87)	−69.00(0.1818) (−170.30~32.30)	−84.14(0.2257) (−220.28~51.99)	−36.11(0.2891) (−102.87~30.66)
18~23 months (n = 303)	Ref.							
24~35 months (n = 435)	25.00(0.6807) (−94.11~144.11)	0.00(1.0000) (−95.78~95.78)	0.00(1.0000) (−71.09~71.09)	0.00(1.0000) (−85.28~85.28)	−60.00(0.1856) (−148.86~28.86)	−39.00(0.4504) (−140.29~62.29)	−75.00(0.2801) (−211.13~61.13)	−16.88(0.6201) (−83.64~49.88)
36~47 months (n = 248)	−65.00(0.3506) (−201.51~71.51)	−150.00(0.0074) (−259.78~−40.22)	−50.00(0.2290) (−131.47~31.47)	−50.00(0.3159) (−147.74~47.74)	−29.37(0.5718) (−131.21~72.47)	−8.00(0.8925) (−124.09~108.09)	0.00(1.0000) (−156.01~156.01)	−53.21(0.1728) (−129.72~23.30)
48~59 months (n = 166)	−125.00(0.112) (−279.16~29.16)	0.00(1.0000) (−123.96~123.96)	−25.00(0.5942) (−117.00~67.00)	−100.00(0.0757) (−210.37~10.37)	−35.00(0.5507) (−150.00~80.00)	−19.00(0.7763) (−150.09~112.09)	25.00(0.7809) (−151.18~201.18)	−30.74(0.4855) (−117.14~55.66)
60~119 months (n = 321)	90.00(0.1732) (−39.53~219.53)	0.00(1.0000) (−104.16~104.16)	25.00(0.5261) (−52.30~102.30)	0.00(1.0000) (−92.73~92.73)	−50.00(0.3104) (−146.63~46.63)	−62.00(0.2698) (−172.15~48.15)	−30.00(0.6911) (−178.03~118.03)	−12.27(0.7404) (−84.86~60.33)
>119 months (n = 75)	90.00(0.4105) (−124.39~304.39)	−50.00(0.5696) (−222.40~122.40)	−25.00(0.7017) (−152.95~102.95)	−150.00(0.0554) (−303.49~3.49)	−15.00(0.8541) (−174.94~144.94)	145.00(0.1190) (−37.32~327.32)	330.00(0.0083) (84.99~575.01)	−19.56(0.7497) (−139.72~100.60)

OLS: ordinary least squares; Q: quantile;

Adjust covariates: Residence, Infant gender, Gestational age, Maternal age, Maternal education, Ethnicity, Gravidity, Maternal job, PIH and Economic status.

For women after a live birth, the OLS estimates showed that, on average, IPI had positive effects on BW between 36 and 59 months; however, OLS did not completely account for the association between the IPI and BW. When we used QR, mothers with an IPI < 12 months had negative effects at the 5^th^ and 10^th^ quantiles of the BW distribution (the lower tail of the distribution), which refers to the very low birth weight (VLBW) group; the OLS showed that there was no significant difference. When the BW was beyond the 90th quantile, mothers with a long IPI (36–59 months) had a significantly lower BW than the reference group.

For the women after a pregnancy termination, multiple linear regression analysis showed that there was no significant difference in BWs between the different IPI and reference group. Nevertheless, there was a statistically significant difference when analysed by the QR. In the VLBW quantiles (10^th^ quantile), it was observed that mothers with IPIs between 36 and 47 months had a negative effect of 150 g on BW compared with the reference group. This finding revealed that mothers with an IPI < 12 months will result in a decrease of 85 g at the 75^th^ quantile, which was significant. The impact of an IPI > 119 months in the upper (95^th^) quantile had an increase of 330 g in BW.

**Figure 1:**
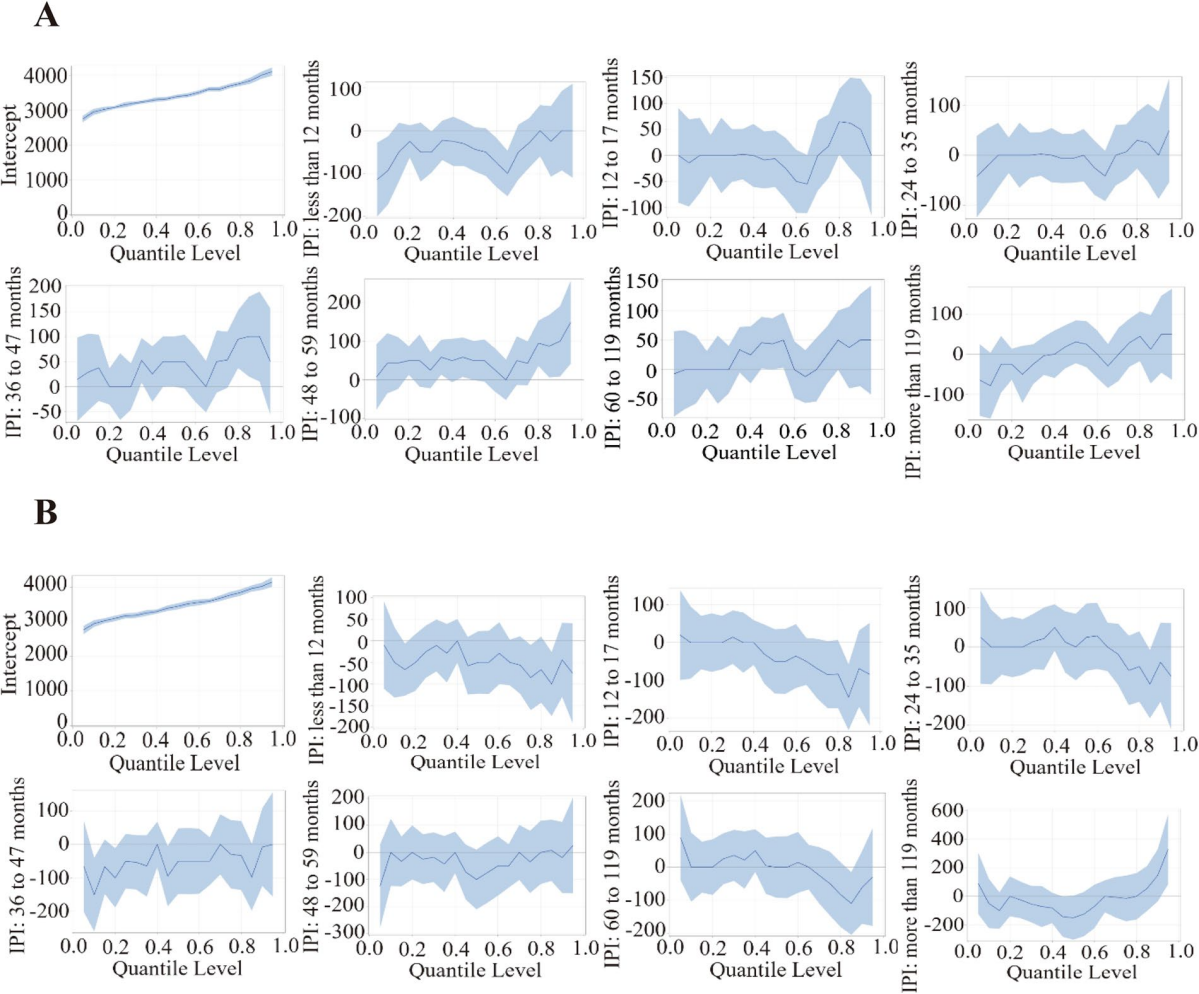
Graphical illustration of QR results of the women after live birth (**A**) and pregnancy termination (**B**), respectively.

The QR model estimates clearly revealed the differential effects of IPIs on the different parts of the BW distribution. Figure 1 showed a graphical illustration of QR results, with all other covariates held constant. The straight solid line in each graph represents the OLS estimates, whereas the curve represents the coefficients of the QR models in the different quantiles of BW and the shadow around the curve shows the 95% confidence band of these estimates.

## Discussion

This paper investigated the association between the IPI and BWs from a large study in Shaanxi Province (western China) between August and October 2013. Our results revealed a clear link between BW and the IPI. QR analysis allowed us to show that the relationships were not uniform across the distribution of BW. The association between a short IPI (<12 months) and BW was greater at smaller quantiles of BW than larger quantiles. The OLS estimation showed that, on average, a long IPI (36~59 months) was related to BW among women after a live birth; however, there was no significant difference among the women after a pregnancy termination. This may be due to the fact that OLS does not completely account for the effect of the IPI on BW. In addition, we found that the IPI among women after a live birth or pregnancy termination had different effects on the BW of the current pregnancy.

Some studies have revealed an apparent optimal IPI of 18~23 months, which is associated with the lowest risk of adverse pregnancy outcomes, including LBW, preterm delivery and small-for-gestational age (SGA)^22,23^. Consequently, our study used an IPI of 18~23 months as the reference. Indeed, IPIs < 12 and >36 months were associated with adverse perinatal outcomes.

Current WHO recommendations are for an IPI of at least 24 months after a live birth^24^. The results of our study corroborated the findings of an earlier study that women with shorter or longer birth intervals are at increased risk of LBW newborns^25^. Women with a short IPI (<12 months) after a live birth are associated with LBW. One of the most studied explanations is related to the nutritional depletion hypothesis, which states that a close succession of pregnancies and period of lactation worsen the mother’s nutritional status and there is insufficient time to restore the energetic and nutritional reserves needed to support fetal growth and development during the subsequent pregnancy^12^. For example, the lack of replenishment of the physiologic depletion of folate that occurs in pregnancy and lactation may lead future pregnancies to be conceived under a state of folate deficiency, thereby increasing the risks of neural tube defects, fetal growth restriction and preterm birth^26^. When the IPI is >36 months, the risk of macrosomia is increased. This finding may due to a long IPI associated with an increased risk of gestational diabetes and entering a pregnancy when obese^27^.

There were different effects on BW between different IPIs among mothers after a live birth or pregnancy termination. After pregnancy termination, our findings were consistent in that a long IPI was associated with LBW. Ball *et al*.^14^ reported that a long IPI was associated with SGA. The increased risk of preterm birth and LBW after a long IPI may in part be because of the co-existing maternal complications, such as pre-eclampsia, hypertension, obesity and diabetes, which are more prevalent as the IPI increases. Another hypothesis for the association between a long IPI and an increased risk of LBW could be explained by a decline in the mother’s physiologic and anatomic adaptation of the reproductive system, which declines gradually after a long time if a woman does not conceive^28^. Both a short IPI (<12 months) and a long IPI (>119 months) were shown to increase the risk of macrosomia. A short IPI provides less time to lose weight from a previous pregnancy, which may increase the likelihood of beginning the next pregnancy obese and the relationship between pre-pregnancy body mass index (BMI) and gestational diabetes^29^. Compared with women with a previous live birth, energy and nutrition status are restored more quickly. In contrast, miscarriage or abortion lengthens the IPI. Also, with older age at the last pregnancy, a longer IPI will be less frequent because of the reduced probability of conception at an older maternal age^30^.

Importantly, IPI is a modifiable risk factor. Indeed, the WHO refers to family planning services as a top priority and not only a key intervention for improving health, but also as a human right^31^. The guidelines on IPIs for women after a termination are of special importance because these women often desire to conceive again with minimal delay. Specifically, a short IPI is desired for some women after a spontaneous abortion or termination due to fetal anomalies and for couples with advanced maternal age or infertility.

Our study had several strengths. The primary strength of the present analysis was the large sample and accurate data. Importantly, our study used QR, a semi-parametric robust regression technique that allows the BW to be treated continuously. The QR has several advantages that applied directly to the analysis of our data set. First, research interest lies in quantiles rather than the means of BWs. Our study interest was the complete distribution of BWs, as follows: LBW; normal; and macrosomia. An inference on the mean BW alone would not be as informative as an inference on multiple quantiles throughout the distribution. Second, the QR has statistical efficiency and is robust with respect to outliers. Outliers and large values have an important effect on the mean and therefore have an impact on linear regression estimates. In contrast, QR is robust to outliers and large values. When the sampled population contains outliers, the quantile estimator is more efficient than the mean estimator for robustness. Third, the QR does not make any request to the stochastic error distribution. This analytic method is important when the distribution is asymmetric and the tail is thick or missing^32^. Compared to OLS, the quantile return has unique superiority in the application. Fourth, the QR does not need to be transformed. There are some transformations, such as the square and the logarithm applied in linear regression. Sometimes the distribution of the dependent variable is skewed and the relationship between independent and dependent variables is non-linear. Converting the outcome may simplify modeling when using linear regression; however, it is often difficult to implement in practice, owing to the inconsistency of back transformation and the challenge of interpretation^33^. In contrast, QR accommodates skewed distributions seamlessly.

Despite the significant implications of these findings, a few limitations of our study are paramount. First, as a result of the limitations of the cross-sectional design, the recall bias could not be eliminated completely, even though we adopted a series of effective measures to control biases as much as possible. Second, the lack of information about some potential confounders, such as maternal pre-pregnancy BMI and placental weight, might be significant factors in the study of IPI and BW, which may in turn increase bias. Third, as our participants were Chinese women of childbearing age from Shaanxi Province in western China, these findings may be limited to the promotion of other ethnic or racial groups. A final limitation of our study was that by using quantile regression, the selection of quantiles was empirically rather than clinically derived^13^.

## Conclusions

Our study showed that IPI is a potentially modifiable factor and both shorter and longer IPIs are independently associated with higher risks of LBW and macrosomia. Our study provides important implications for reducing LBW and macrosomia. Optimization of the IPI is indicated for good pregnancy outcomes. Healthcare providers should counsel women who have recently given birth regarding the risk to subsequent pregnancy and the adverse effect of shorter or longer IPIs on subsequent birth. The importance of providing support for family planning services should be emphasized and the suggestion of an optimal IPI should be included to reduce adverse birth outcomes.
